# Development and validation of a questionnaire for the knowledge assessment and management of PLADO diet in kidney and healthy population in Cyprus

**DOI:** 10.3389/fnut.2025.1619237

**Published:** 2025-07-04

**Authors:** Anna Michail, Constandinos Zesimos, Iris Sergiou, Katerina Tzini, Eleni Andreou

**Affiliations:** Department of Life Sciences, School of Life and Health Sciences, University of Nicosia, Nicosia, Cyprus

**Keywords:** chronic kidney disease, protein perception, questionnaire validation, plant-based diet, sustainability, renal nutrition, dietary education, Cyprus

## Abstract

Chronic kidney disease (CKD) requires dietary strategies that balance protein restriction, nutritional adequacy, and sustainability. As plant-dominant dietary models gain prominence in renal nutrition, understanding public perceptions of protein sources and their health impacts is increasingly critical. However, no validated assessment tool exists to evaluate such perceptions within the Cypriot population. This study presents the development and validation of a novel questionnaire designed to assess perceptions of sustainability, nutritional value, and health impacts of plant- and animal-based proteins, with a focus on kidney health. The instrument was developed through literature review, expert input (*n* = 10), cognitive pretesting, and pilot testing (*n* = 120). Validation included content validity indexing (I-CVI), Modified Kappa statistics, internal consistency via Cronbach's alpha, and exploratory factor analysis (EFA). Expert agreement was high, with an S-CVI/Ave of 0.89 and 93% of items achieving I-CVI ≥ 0.83. Internal consistency across subscales ranged from α = 0.71 to 0.82. EFA supported construct validity, explaining 36% of the variance. The final 42-item questionnaire covers eight domains, including sustainability beliefs, dietary behavior, and protein knowledge. A unique feature is its embedded educational content—glossary terms, visuals, and explanatory prompts—designed to evaluate baseline knowledge and learning outcomes. Pilot results revealed knowledge gaps and uncertainty about the sustainability of plant-based proteins among CKD respondents. This validated tool fills a significant gap in renal nutrition education and research and offers a reliable, culturally relevant means to assess dietary perceptions. It supports patient education, public health interventions, and clinical practice in promoting sustainable, kidney-friendly diets. Broader application and cross-cultural adaptation are recommended to enhance global utility.

## 1 Introduction

Understanding consumer perceptions of sustainability, nutrition, and the health impact of dietary protein sources is increasingly relevant in the context of evolving dietary guidelines and public health strategies. In particular, individuals with chronic kidney disease (CKD) require careful dietary management, especially regarding protein intake, to maintain kidney function and overall wellbeing. CKD affects over 10% of the global population and is frequently underdiagnosed (1). Its prevalence increases significantly with age—affecting 42% of those over 75, 21% of individuals aged 65–74, and 6% among those aged 18–54. CKD is commonly comorbid with cardiovascular disease and diabetes, further increasing morbidity and mortality risk (2).

While animal-based proteins have traditionally been the primary dietary source, recent trends emphasize the potential health and environmental benefits of plant-based proteins. Although existing research explores the physiological effects of various protein sources in CKD management, limited attention has been given to how consumers perceive these dietary alternatives—particularly in specific populations such as Cypriots with or at risk of CKD. Given that dietary behaviors are shaped by knowledge, attitudes, and beliefs (3), it is crucial to develop valid tools to assess consumer perceptions regarding sustainability and the health impacts of plant- and animal-based proteins.

The global rise in chronic kidney disease (CKD), linked with metabolic disorders, calls for early intervention strategies grounded in nutritional prevention. Plant-based, low-protein dietary patterns are increasingly recognized for their potential to delay CKD progression and reduce cardiovascular risk (4). However, adherence remains low, often due to cultural beliefs, knowledge gaps, and concerns over nutritional adequacy. The Plant-Dominant Low-Protein Diet (PLADO) framework offers a structured approach emphasizing plant protein, portion control, and sustainability—yet its adoption requires targeted educational tools. Sustainable dietary interventions not only support individual health but also align with global environmental goals (5, 6). Thus, a validated questionnaire is needed to measure perceptions across both health and ecological dimensions, particularly in culturally diverse settings.

In Cyprus, a Mediterranean country with diverse dietary practices, food choices are influenced by cultural norms, environmental awareness, and evolving dietary trends (7). A recent review highlights the potential for plant-dominant low-protein diets (PLADO) as a culturally relevant and clinically effective approach for CKD management in Cyprus, integrating both traditional cuisine and nutritional science (5). As public interest in sustainable eating grows, it becomes essential to understand how Cypriots perceive the interplay between diet, health, and environmental responsibility (8). This is particularly relevant for individuals with CKD, whose dietary habits can significantly influence disease progression and quality of life. Despite increased awareness of protein sustainability and its health implications, no validated tool currently exists to assess such perceptions among Cypriot populations.

To address this gap, we developed a structured questionnaire designed to evaluate beliefs, attitudes, and knowledge related to sustainable dietary practices and the health implications of protein choices, with a focus on CKD. The questionnaire integrates educational content—such as brief definitions and illustrated food comparisons—to evaluate baseline understanding and the potential effect of targeted nutrition education. This dual approach facilitates the assessment of both pre-existing knowledge and post-intervention perception shifts.

Although Cyprus shares many characteristics with Mediterranean dietary patterns—such as olive oil use, fresh vegetables, and moderate wine consumption—it also exhibits distinct regional features. Notably, traditional Cypriot cuisine includes a high intake of pork products, halloumi cheese, and grilled meats (8, 9), setting it apart from the plant-rich diets of other Mediterranean populations like Greece or Italy. Additionally, plant-dominant dietary models are less culturally ingrained in Cyprus, where meat consumption remains a central part of communal meals and festivals (10). These cultural differences underscore the importance of developing a regionally adapted questionnaire to accurately capture perceptions of protein sources and their health and sustainability implications within the Cypriot context.

In addition to health considerations, the sustainability of dietary choices is increasingly important—especially for individuals with CKD, who require specific protein intake adjustments. Plant-based proteins, when compared to animal sources, are associated with lower greenhouse gas emissions, reduced land and water use, and lower ecological burden. Mediterranean populations, including Cypriots, are well-positioned to adopt plant-dominant low-protein diets (PLADO) due to traditional food patterns that emphasize legumes, grains, and seasonal produce. Integrating sustainability into CKD nutrition offers a dual benefit: mitigating disease progression and supporting environmentally responsible eating practices.

The primary objective of this study is to develop and validate a questionnaire that captures consumer perceptions and knowledge related to sustainable dietary practices, with specific focus on early-stage CKD prevention and management. This questionnaire was developed for use in both the general Cypriot population and individuals diagnosed with early to moderate stages of CKD (stages 1–3), based on self-reported or clinician-confirmed eGFR data. Individuals on dialysis or those with kidney transplants were excluded from the study. The instrument is intended to support both the prevention of CKD progression through education and the early-stage dietary management of the disease via sustainable and renal-appropriate protein choices. The tool is designed to explore three main domains:

First, it examines consumer understanding of sustainability in relation to protein sources. With mounting concerns about environmental impact and food system sustainability, it is important to evaluate how well consumers recognize these factors in their food choices.

Second, it assesses awareness of the nutritional differences between plant-based and animal-based proteins in the context of CKD management. Excess protein intake, particularly from certain animal sources, can accelerate CKD progression. Evaluating consumer knowledge in this area informs education strategies.

Third, the questionnaire explores how dietary protein choices are perceived to affect kidney function and health. For individuals at risk of or living with CKD, it is vital to assess their understanding of the potential health consequences of different protein sources.

A unique feature of the tool is the integration of evidence-based educational content, including visual guides and simplified definitions to enhance comprehension. This approach enables evaluation of baseline knowledge and post-intervention learning, identifying gaps and misconceptions regarding protein intake, sustainability, and CKD.

Validation ensures the questionnaire reliably captures both baseline knowledge and changes following educational exposure. The full validation framework, including content relevance, clarity, and construct structure, is described in detail in the Methods section.

To address the research gap and support culturally relevant dietary interventions, the scope of the study was defined as follows. This study aimed to develop and validate a culturally appropriate questionnaire assessing consumer perceptions of sustainability, nutritional knowledge, and health impacts of dietary protein sources—specifically targeting individuals at risk of or living with chronic kidney disease (CKD) in Cyprus. The instrument is intended for use in public health education, clinical nutrition counseling, and future research focused on plant-dominant low-protein diets (PLADO) and kidney health.

## 2 Methods and materials

### 2.1 Questionnaire development

The questionnaire was developed to assess three primary constructs: sustainability perceptions, dietary habits, and awareness of health impacts related to protein intake, particularly among individuals at risk of or living with chronic kidney disease (CKD). Constructs were identified through an extensive literature review on plant-based and animal-based protein consumption and their effects on kidney health. Development procedures followed recommended best practices for health-related scale creation, including guidelines proposed by Boateng et al. (11), Ranganathan et al. (12), and the COSMIN checklist for content validity.

#### 2.1.1 Questionnaire domains and objectives

The questionnaire was structured around four domains:

Sustainability Perceptions—assessed awareness of environmental impacts of protein sources, including greenhouse gas emissions, water usage, and ethical considerations.

*Example item:* “A plant-based or vegetarian diet yields less meat, less greenhouse gas emissions, more love for the planet's animals, less waste of water and land... Can vegetable proteins be considered a viable alternative?”

Dietary Habits—included 14 items modeled on the MedScore framework and dietary classification systems (e.g., vegan, DASH, PLADO). Food frequency items measured plant- vs. animal-protein intake.

*Example item:* “How frequently do you consume legumes?”

Health Impacts—evaluated knowledge of how protein choices affect weight management, kidney function, and clinical biomarkers.

*Example item:* “Which type of protein do you think can negatively affect kidney function when consumed in large quantities over time?”

Knowledge Assessment—examined understanding of protein roles in the body and nutrient composition.

*Example item:* “Do you know how many grams of protein one slice of white bread contains?”

This domain structure ensured comprehensive assessment of perceptions, habits, and knowledge surrounding sustainable, kidney-friendly protein choices. Supplementary Table 1 provides representative items from each domain of the questionnaire, illustrating the thematic focus and assessment scope used to evaluate sustainability beliefs, dietary habits, protein knowledge, and health perceptions relevant to CKD.

#### 2.1.2 Item generation process

Item generation was informed by a systematic review of the literature conducted through PubMed and the University of Nicosia Library databases. Search terms included: (Animal protein OR vegetarian protein OR plant-based) AND (Health populations OR Kidney patients) AND (Questionnaire OR Tool) AND (Health impact). From 84 articles screened, 11 utilized questionnaires, and only two involved the development of knowledge-based assessment tools (13, 14).

Items were designed iteratively in collaboration with domain experts in nutrition, nephrology, and public health. Educational materials, including visual aids, glossary definitions, and culturally adapted language examples, were incorporated to enhance participant comprehension.

#### 2.1.3 Initial questionnaire design and cognitive pretesting

The initial draft comprised five sections:

- Demographics and health history (including kidney status and biomarker history)
- Food frequency for vegetarian and animal protein consumption
- Protein functions and nutrient knowledge
- Mediterranean Diet adherence scoring (MedScore; Yes/No format)
- Perceptions of sustainability

A cognitive pretesting phase was conducted with ten undergraduate nutrition students to evaluate face validity and comprehension. Feedback led to simplifications in terminology, enhanced visual supports, and inclusion of culturally relevant food examples (e.g., lentils with rice).

### 2.2 Questionnaire validation process

#### 2.2.1 Content and face validity

Content validity was evaluated by a panel of nine domain experts (nutrition, nephrology, dietetics), who independently rated each item's relevance, clarity, and simplicity using a 5-point Likert scale. Item-level Content Validity Index (I-CVI) scores were calculated, with I-CVI ≥0.78 considered acceptable. Scale-level CVI (S-CVI/Ave) was also computed to assess overall coverage. Modified Kappa statistics were applied to adjust for chance agreement, interpreted as ≥0.74 (excellent), 0.60–0.74 (good), 0.40–0.59 (fair), and < 0.40 (poor).

Face validity was further assessed during cognitive pretesting, leading to refinements in item clarity, visual formatting, and educational materials.

#### 2.2.2 Construct validity (exploratory and confirmatory factor analysis)

Construct validity was explored using Exploratory Factor Analysis (EFA) based on Classical Test Theory. Factors were extracted using eigenvalues >1, scree plot evaluation, and theoretical interpretability. A minimum factor loading of 0.40 was used for item retention. Confirmatory Factor Analysis (CFA) was performed subsequently to validate the factor structure, with model fit evaluated via indices such as RMSEA, TLI, and BIC.

#### 2.2.3 Internal consistency and reliability testing

Internal consistency reliability was assessed using Cronbach's alpha, with α ≥ 0.70 considered acceptable. Additional item-level analyses included:

- Missing value analysis (15).
- Critical value analysis (16).
- Item-total correlation assessment (17).
- Homogeneity testing (18).

Although internal consistency was assessed, test–retest reliability was not performed in this phase and is recommended for future validation.

#### 2.2.4 Criterion validity assessment

Criterion validity was assessed through correlations between the MedDietScore-derived dietary adherence results and scores obtained from the validated Mediterranean Diet Adherence Screener (19).

### 2.3 Pilot testing procedures

#### 2.3.1 Cognitive pilot study (*n* = 10)

A cognitive validation process was conducted with 10 participants to assess item clarity, relevance, and interpretability. The expert panel included one biostatistician, eight academic professionals and clinical dietitians with specialization in kidney nutrition, and one patient with chronic kidney disease. Experts were invited via formal email communication and participated by independently reviewing the questionnaire through an online Google Form. They rated each item for clarity, importance, and simplicity using a structured scale. Feedback from this panel informed refinements to item phrasing, educational glossaries, and scoring instructions. This procedure aligns with established best practices in instrument development and content validation (20).

#### 2.3.2 Field psychometric pilot study (*n* = 120)

A subsequent field pilot study was conducted with 120 adult participants recruited through nephrology clinics and public advertisements. Inclusion criteria were: age ≥18 years, Greek-speaking, and internet access. The questionnaire used in this phase consisted of 42 items organized across 8 thematic domains, including sustainability beliefs, dietary habits, CKD knowledge, and protein-related behaviors. Exclusion criteria included dialysis dependence, kidney transplantation, or cognitive impairment. Participants completed the 42-item questionnaire administered via Google Forms. Data collected included demographics, dietary habits, health history, and protein knowledge. CKD diagnosis was determined based on self-reported medical history and confirmed through documented eGFR values, as captured in the questionnaire (Data Sheet 1, Question 14). Participants who reported dialysis dependence or kidney transplantation were excluded from the analysis.

### 2.4 Data analysis

Statistical analyses were performed using Python-based libraries: pandas, scipy.stats, and statsmodels for reliability analyses, and sklearn for confirmatory factor analyses. Visualizations were created using matplotlib and seaborn. Scoring procedures included the summation of correct responses for knowledge domains, binary coding for Mediterranean Diet adherence, and frequency-based categorization for sustainability and protein intake patterns scoring procedures were standardized:

- Mediterranean Diet Score (14 dichotomous Yes/No items).
- Knowledge scores (sum of correct responses).
- Sustainability and protein perceptions (categorical variables).

All procedures involving human participants were approved by the Cyprus National Bioethics Committee (Protocol number EEBK E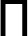 2024.01.53).

## 3 Results

### 3.1 Content validity

The content validity of the questionnaire was evaluated by a panel of nine domain experts specializing in nutrition, nephrology, and dietetics. Each item was assessed for relevance, clarity, and simplicity using a 5-point Likert scale. Item-Level Content Validity Index (I-CVI) scores were calculated, with 93% of items achieving an I-CVI ≥0.83 and 68% attaining a perfect score of 1.00. The Scale-Level CVI (S-CVI/Ave) was 0.89, indicating excellent overall agreement on content relevance. Modified Kappa statistics, adjusting for chance agreement, demonstrated that most items fell within the “excellent” (κ ≥ 0.74) or “good” (κ = 0.60–0.74) categories. These findings substantiate the strong content validity of the questionnaire. Full I-CVI and Modified Kappa statistics are presented in Supplementary Table 2.

### 3.2 Face validity

Face validity was assessed through cognitive pretesting with a small pilot group of 10 undergraduate nutrition students. Participants provided feedback on item clarity, terminology, and conceptual understanding, particularly related to protein knowledge. Based on the feedback, modifications were made to simplify definitions, incorporate additional educational visuals, and enhance the accessibility of technical concepts. These revisions improved the comprehensibility and usability of the instrument.

### 3.3 Internal consistency and reliability

Internal consistency reliability of the questionnaire was evaluated using Cronbach's alpha across key subscales. The MedDietScore subscale (14 binary items) demonstrated good reliability (α = 0.82). The Sustainability Beliefs subscale showed acceptable reliability (α = 0.76), and the CKD Protein Knowledge subscale also achieved acceptable consistency (α = 0.71). The Food Frequency subscale exhibited borderline acceptable internal consistency (α = 0.69), suggesting potential areas for future refinement. Full internal consistency statistics are provided in Supplementary Table 2.

### 3.4 Construct validity

Construct validity was assessed through exploratory factor analysis (EFA). Factors were extracted using eigenvalues >1 and scree plot examination. Factor loadings exceeded 0.40 for most items, supporting the presence of coherent latent constructs. The dominant factor explained 36% of the total variance, supporting the structural validity of the questionnaire. Confirmatory factor analysis (CFA) further supported model adequacy, although improvements could enhance fit indices in future refinements. Details of factor analysis results are presented in Supplementary Table 3.

### 3.5 Pilot study demographics

A field pilot study was conducted with 120 adult participants to evaluate demographic representation and psychometric performance. The sample had a balanced gender distribution (52.5% male, 47.5% female), with a mean age in the late twenties, and the majority possessing tertiary education qualifications (92.5%). Employment status was diverse, comprising 60% employed individuals and 30% students. Full demographic characteristics are provided in Supplementary Table 4.

### 3.6 Pilot sample CKD stratification and scores

The pilot study included 120 participants, of whom 35.8% (*n* = 43) self-reported having chronic kidney disease (CKD). Based on available data, CKD stages were distributed as follows: Stage 1 (eGFR ≥ 90)–12%, Stage 2 (eGFR 60–89)–14%, and Stage 3a−3b (eGFR 30–59)–9.8%. Individuals with an eGFR below 30, those undergoing dialysis, or who had received a kidney transplant were excluded from the sample.

Participants with CKD scored slightly lower on the protein knowledge scale (Mean = 1.67, SD = 1.39) compared to those without CKD (Mean = 2.11, SD = 1.24). The overall mean Mediterranean Diet Score (MedDiet Score) was 7.75 ± 2.38, reflecting moderate adherence to Mediterranean dietary principles. Perceptions around sustainability—especially plant-based protein adequacy—were more variable among CKD participants, with many expressing uncertainty regarding nutritional sufficiency. These comparative results are summarized in Supplementary Table 4.

## 4 Discussion

This study presents the development and psychometric validation of a novel questionnaire designed to assess perceptions of sustainability, nutritional knowledge, and health impacts of plant- and animal-based protein sources, with a particular emphasis on kidney health. The questionnaire demonstrated strong content validity, acceptable to good internal consistency across subscales, and promising construct coherence. These findings support its utility as a reliable and culturally tailored instrument for assessing dietary perceptions within the Cypriot population.

The content validation process showed high expert consensus, with a scale-level content validity index (S-CVI/Ave) of 0.89 and most items reaching excellent or good agreement in Modified Kappa statistics. Face validity testing through cognitive pretesting further improved the clarity and comprehensibility of the instrument, particularly by refining educational components and technical terminology. Feedback from this pretesting phase and subsequent modifications are summarized in Supplementary Table 5.

Internal consistency reliability, assessed via Cronbach's alpha, was acceptable across most subscales (α = 0.71–0.82), consistent with established thresholds for health-related questionnaires. Specifically, the MedDietScore and Sustainability Beliefs subscales exhibited strong reliability, while the Protein Source Frequency subscale demonstrated borderline acceptability, suggesting potential areas for future refinement. As shown in Supplementary Table 6, internal consistency varied across subscales. The *MedDietScore* subscale achieved the highest reliability (α = 0.82), followed by *Sustainability Beliefs* (α = 0.76). Lower alpha values in the *24-h Recall Consistency* (α = 0.65) and *Protein Source Frequency* (α = 0.69) subscales suggest potential item heterogeneity and indicate areas for future refinement.

Construct validity was supported by exploratory factor analysis (EFA), which revealed a dominant clarity-related factor explaining 36% of the total variance. Factor loadings for most items exceeded 0.40, indicating a coherent underlying structure. Detailed EFA results, including factor loadings and variance explained, are provided in Supplementary Table 7. Confirmatory factor analysis (CFA) further explored model fit, with indices such as RMSEA and BIC supporting structural adequacy, although the Tucker-Lewis Index (TLI) suggested opportunities for model improvement.

Moreover, inter-item reliability among expert ratings of relevance, clarity, and simplicity was high, with standardized Cronbach's alpha values exceeding 0.90, reinforcing the robustness of the content validation phase (Supplementary Table 8).

In addition to the psychometric properties, the discussion now emphasizes pilot results more directly. Participants with CKD scored lower on protein knowledge and expressed greater uncertainty around the sustainability of plant-based diets—particularly the sufficiency of plant proteins for kidney health. These findings suggest knowledge gaps that may undermine adherence to recommended low-protein dietary regimens such as PLADO.

While the PLADO framework has gained attention for its potential to delay CKD progression and reduce cardiovascular burden, recent literature also raises concerns regarding its nutritional adequacy—particularly for *protein and* micronutrient sufficiency in advanced CKD stages (21, 22). These findings highlight the need for evidence-based educational interventions to guide patients toward safe implementation. Our questionnaire aims to fill this gap by evaluating both the perceived risks and informational gaps that may hinder safe adherence.

Perceptions of sustainability varied substantially, with many CKD participants unsure about the environmental or nutritional adequacy of plant-based options. This reinforces earlier findings from Mediterranean and Cypriot contexts, where plant-based transitions face cultural and informational barriers. Our tool is the first to explicitly integrate both health and environmental dimensions of protein intake in the context of kidney disease.

Comparison with existing instruments shows that while tools such as the MedDietScore assess overall dietary quality, they lack integration of sustainability or kidney-specific considerations. Our instrument bridges this gap and enables targeted interventions through clinical and community settings.

Furthermore, the suggested tool incorporates visual elements—such as portion-size diagrams and annotated glossaries—to improve comprehension and dietary self-efficacy. This design feature aligns with recent findings demonstrating that visual aids enhance adherence to healthy dietary patterns, particularly in CKD populations (23–25). These elements strengthen the questionnaire's utility not only as a perception assessment tool but also as a behaviorally-informed educational intervention that may be applied in both clinical and community settings.

The questionnaire is structured for adaptability and future global use. Cross-cultural validation, digital deployment, and translation will be key next steps. The tool is positioned to support both individual-level dietary counseling and broader public health strategies—especially relevant in regions with rising CKD prevalence and shifts toward sustainable diets (26–32).

Several strengths characterize this study. The multi-phase validation approach—spanning expert evaluation, cognitive pretesting, field pilot testing, and psychometric assessment—strengthens the scientific rigor and credibility of the findings. Additionally, the incorporation of educational content to measure baseline knowledge and post-assessment learning represents an innovative and practical advancement (5, 27–35).

Nonetheless, several limitations should be acknowledged. First, the pilot sample predominantly consisted of younger adults with higher education levels, potentially limiting generalizability to other population groups (36–45). Second, test–retest reliability was not assessed in this phase and should be incorporated into future longitudinal validation efforts (46–57). Third, while preliminary construct and criterion validity findings were promising (Supplementary Table 9), further validation against clinical outcomes, such as biomarkers of kidney function, is necessary to confirm predictive validity (10, 13, 58–68).

Future research should focus on confirming the questionnaire's temporal stability through test–retest reliability studies, evaluating its predictive validity in CKD progression, and conducting cross-cultural adaptation studies to enhance broader applicability. Furthermore, applying the tool in different CKD stages and in general population cohorts could provide deeper insights into dietary behavior modification strategies and public health interventions (69–85).

Finally, this validated questionnaire offers a culturally relevant, psychometrically sound tool to support clinical practice, public health initiatives, and research efforts aimed at promoting sustainable and kidney-friendly dietary behaviors (5, 11, 86–90).

## 5 Conclusion

This study presents a rigorously developed and validated questionnaire that uniquely integrates sustainability, nutritional knowledge, and kidney-specific dietary principles. The instrument demonstrates strong psychometric properties and cultural relevance, particularly for Cypriot and Mediterranean populations. Its potential for cross-cultural adaptation, digital deployment, and clinical use positions it as a valuable tool for promoting informed, sustainable dietary choices among individuals at risk for or living with CKD.

## Data Availability

The raw data supporting the conclusions of this article will be made available by the authors, without undue reservation.
