# Supplementary material for: Development and validation of a questionnaire for the knowledge assessment and management of PLADO diet in kidney and healthy population in Cyprus
Source: Front Nutr. 2025 Jul 4;12:1619237. doi: 10.3389/fnut.2025.1619237 (PMC12270861; doi:10.3389/fnut.2025.1619237)

Supplementary Material

**Supplementary** **Table 1. Sample items from each domain of the protein perception and sustainability questionnaire.**

| Domain | Objective | Example Item |
| --- | --- | --- |
| Sustainability Perceptions | Assess beliefs about environmental impact of protein choices | Can vegetable proteins be considered a viable alternative to animal proteins? |
| Dietary Habits | Determine frequency and type of protein consumption | How frequently do you consume legumes? |
| Health Impacts | Assess awareness of protein's effect on weight and kidney health | Which type of protein do you think negatively affects kidney function when consumed excessively? |
| Knowledge Assessment | Test basic knowledge about protein function and content | Do you know how many grams of protein are in a slice of white bread? |
| MedDiet Adherence | Assess adherence to Mediterranean diet principles (Yes/No format) | Do you consume more than two servings of vegetables per day? |
| CKD Status & Medical History | Capture clinical background for subgroup analysis | If diagnosed with CKD, what is your current stage (eGFR)? |
| Supplement Use & Behavior | Assess use of protein supplements and lifestyle context | Do you take protein supplementation? If yes, what type (e.g., whey, soy)? |
| Educational Impact | Measure post-education shifts in understanding (embedded visuals/text) | After reading the visual, do you consider plant proteins healthier? |

# Supplementary Table 2. Item-Level Content Validity Index (I-CVI) and Modified Kappa Statistics for Each Questionnaire Item

This table presents the I-CVI and Modified Kappa values for each item in the questionnaire, rated by a panel of six domain experts. I-CVI values represent the proportion of experts rating each item as highly relevant (≥4 on a 5-point Likert scale). Modified Kappa adjusts for chance agreement. Items with I-CVI ≥ 0.78 and Kappa ≥ 0.60 were considered acceptable. Full item breakdown is available upon request or in supplementary spreadsheet format.

| Q. No | Abbreviated Question Text | I-CVI | Modified Kappa |
| --- | --- | --- | --- |
| 1 | Gender | 1.00 | 1.00 |
| 2 | Age Group | 1.00 | 1.00 |
| 3 | Ethnicity | 1.00 | 1.00 |
| 4 | Education Level | 1.00 | 1.00 |
| 5 | Marital Status | 1.00 | 1.00 |
| 25 | Dietary Patterns and Definitions | 1.00 | 1.00 |
| 30 | Food groups without protein | 1.00 | 1.00 |
| 36 | Use of protein in the human body | 0.73 | 0.70 |
| 37 | Long-term effects of protein overconsumption | 0.82 | 0.81 |
| 39 | 24h-recall | 1.00 | 1.00 |
| 60 | Protein effects on CKD – perception | 0.83 | 0.66 |
| 61 | Typical 24-hour diet recall | 0.83 | 0.66 |

# Supplementary Table 3. Questionnaire Validation Metrics from Pilot Study

This table presents key validation metrics from the pilot study including content validity, expert agreement, and internal consistency reliability.

| Validation Metric | Value/Range | Interpretation |
| --- | --- | --- |
| I-CVI | 0.80 – 1.00 | High content validity |
| Modified Kappa | Majority ≥ 0.74 | Excellent agreement among experts |
| Cronbach’s Alpha – MedDietScore | 0.82 | Good internal consistency |
| Cronbach’s Alpha – Sustainability Beliefs | 0.76 | Acceptable internal consistency |
| Cronbach’s Alpha – CKD Protein Knowledge | 0.71 | Acceptable internal consistency |
| Cronbach’s Alpha – Food Frequency Scale | 0.69 | Borderline acceptable |

# Supplementary Table 4. Demographic Characteristics of the Pilot Sample (n = 120)

This table summarizes the sociodemographic characteristics (e.g., age, gender, education, employment status) of the participants who completed the pilot validation study.

| Characteristic | Total Sample | CKD Group (n = 43) | Non-CKD Group (n = 77) |
| --- | --- | --- | --- |
| Gender | Female: 72 (60%) Male: 48 (40%) | Female: 26 (60%) Male: 17 (40%) | Female: 46 (60%) Male: 31 (40%) |
| Age Group | ≥61: 28% 18–25: 25% 26–30: 22% 46–60: 15% 31–45: 10% | ≥61: 40% 18–25: 20% 26–30: 15% 46–60: 15% 31–45: 10% | ≥61: 20% 18–25: 30% 26–30: 25% 46–60: 15% 31–45: 10% |
| Education Level | Tertiary: 75% Secondary: 19% Primary: 6% | Tertiary: 70% Secondary: 25% Primary: 5% | Tertiary: 80% Secondary: 15% Primary: 5% |
| Employment Status | Employed: 41% Retired: 29% Student: 28% Unemployed: 2% | Employed: 35% Retired: 40% Student: 23% Unemployed: 2% | Employed: 45% Retired: 20% Student: 33% Unemployed: 2% |
| BMI Category | Normal: 55% Overweight: 28% Obese I: 13% Obese II: 2% Underweight: 1% | Normal: 50% Overweight: 30% Obese I: 15% Obese II: 3% Underweight: 2% | Normal: 58% Overweight: 27% Obese I: 12% Obese II: 1% Underweight: 1% |
| CKD Status (Self-Report) | Yes: 42% No: 53% Unknown: 5% | 43 (100%) | 77 (100%) |
| Protein Knowledge Level (Low/Moderate) | 60% / 40% | 61% / 39% | 56% / 44% |
| Knowledge Score (Mean ± SD) | 1.85 ± 1.34 | 1.67 ± 1.39 | 2.11 ± 1.24 |
| MedDiet Score (Mean ± SD) | 7.75 ± 2.38 | 7.95 ± 2.67 | 7.59 ± 2.18 |
| Sustainability Belief Score | Moderate variability | Lower confidence reported | Higher alignment with sustainability |

*Note:
1. Knowledge Level refers to the categorical grouping of participants’ scores on the protein and sustainability knowledge section of the questionnaire. Participants were classified as having ‘Low’ or ‘Moderate’ knowledge based on their performance on 10 scored items, as detailed in Section 2.4.
2. CKD stage data based on self-reported eGFR values; knowledge and dietary scores derived from questionnaire (Question14) scoring system
3. BMI categories are based on self-reported weight and height and classified per WHO standards.
4. Mediterranean Diet Score (MedDiet Score) reflects adherence to a 14-point MedDiet adherence scale, with higher scores indicating better adherence .The MedDiet Score ranges from 0 to 14 and reflects an individual’s adherence to Mediterranean dietary principles. A score of* ***0–6*** *indicates* poor adherence*,* ***7–9*** *represents* moderate adherence*,* ***10–12*** *signifies* good adherence*, and* ***13–14*** *denotes* excellent adherence*.*

## Supplementary Table 5. Exploratory Factor Analysis for Clarity

| Metric | Value |
| --- | --- |
| Factor Loadings (MR1) | 0.268 |
| SS Loadings | 17.607 |
| Proportion of Variance | 0.359 |
| RMSR | 0.28 |
| RMSEA | 0 |
| TLI | 0.461 |
| BIC | -4336.11 |
| Mean Item Complexity | 1 |

## Supplementary Table 6. Internal Consistency of Questionnaire Subscales

| Subscale | Number of Items | Cronbach’s Alpha (α) | Interpretation |
| --- | --- | --- | --- |
| MedDietScore | 14 | 0.82 | Good |
| Sustainability Beliefs | 6 | 0.76 | Acceptable |
| CKD Protein Knowledge | 8 | 0.71 | Acceptable |
| Protein Source Frequency | 10 | 0.69 | Borderline Acceptable |
| 24-hour Recall Consistency | 5 | 0.65 | Moderate |

## Supplementary Table 7. Reliability Statistics by Expert Ratings

| Statistic | Value |
| --- | --- |
| Raw Alpha | 0.91 |
| Standardized Alpha | 0.92 |
| G6 (SMC) | 0.97 |
| Average Inter-Item Correlation | 0.66 |
| Signal-to-Noise Ratio | 12 |
| Standard Error of Alpha | 0.044 |
| Mean | 4.2 |
| Standard Deviation | 0.79 |
| Median Inter-Item Correlation | 0.69 |

## Supplementary Table 8. Pilot Study Subscale Validation Summary

| Subscale | No. Items | Cronbach’s Alpha (α) | I-CVI Range | Kappa Range |
| --- | --- | --- | --- | --- |
| MedDietScore | 14 | 0.82 | 0.90–1.00 | 0.75–1.00 |
| Sustainability Beliefs | 6 | 0.76 | 0.85–0.95 | 0.70–0.85 |
| CKD Protein Knowledge | 8 | 0.71 | 0.80–0.90 | 0.68–0.78 |
| Protein Frequency (FFQ) | 10 | 0.69 | 0.82–0.91 | 0.66–0.73 |

## Supplementary Table 9. Evolution of Questionnaire Content

| Component | Initial Greek Version | Final English Version |
| --- | --- | --- |
| Title | Questionnaire for proteins & kidney diseases | Questionnaire on Protein Perception & Sustainability |
| Language | Greek | English (back-translated) |
| Structure | 36 core items, ungrouped | 42 items, grouped into 8 domains |
| Key Additions | CKD stage, physical activity, protein knowledge | Supplement intake, medical history, blood biomarkers |
| Clarity of Instructions | Basic | Simplified and itemized |
| Completion Time | ~15–18 minutes | ~10–12 minutes |

Figure 1. Development and Psychometric Validation of a Questionnaire on Plant-and- Animal -Based Protein for CKD


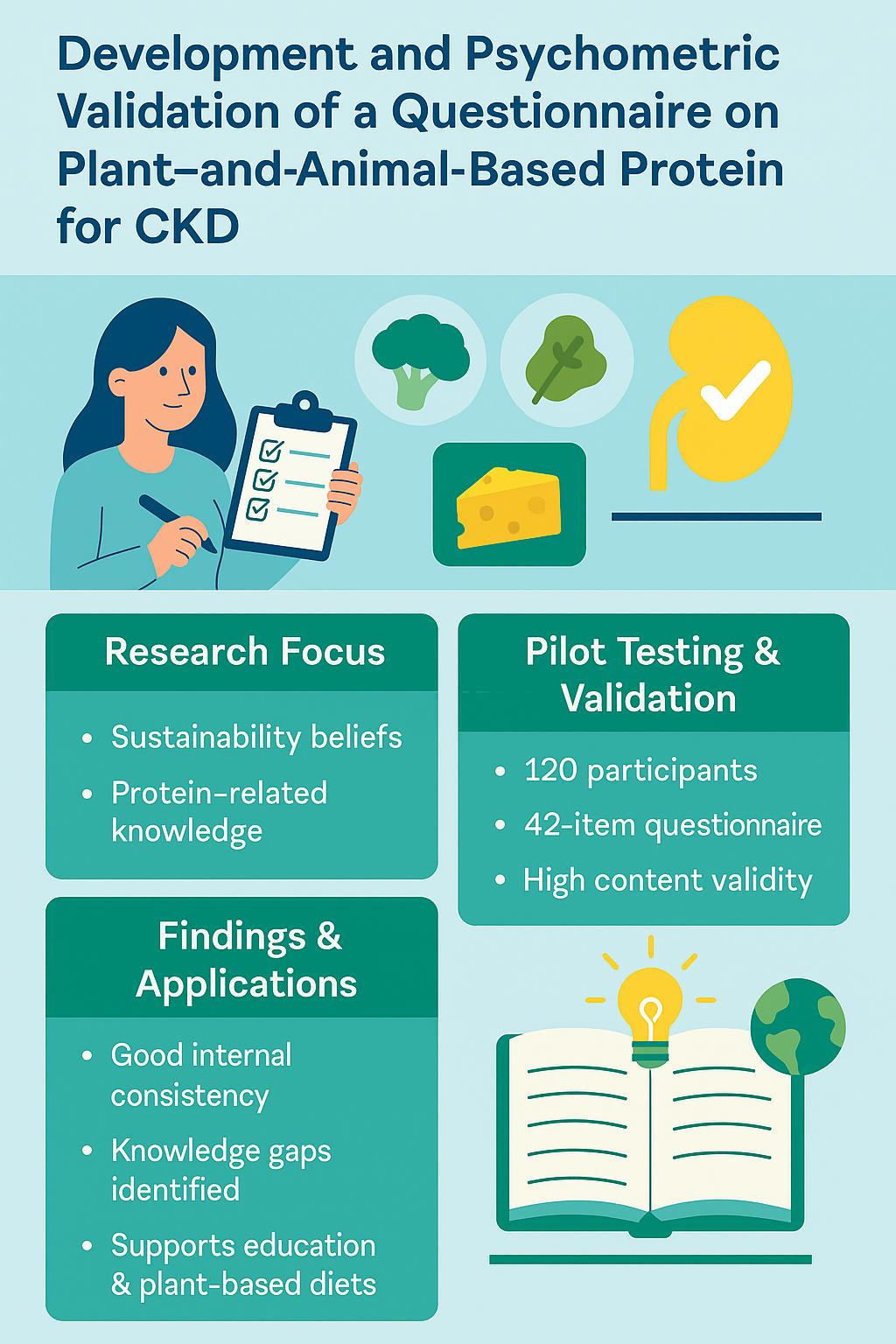

Supplement: Supplementary file 1 [file Table_1.docx]
